# Measuring effects of screen time on the development of children in the Philippines: a cross-sectional study

**DOI:** 10.1186/s12889-023-16188-4

**Published:** 2023-06-28

**Authors:** Angel Belle C. Dy, Alane Blythe C. Dy, Samantha Katrina Santos

**Affiliations:** Ateneo de Manila University, School of Medicine and Public Health, Ortigas Avenue, Pasig City, 1604 Philippines

**Keywords:** Child development, Screen time, Language development, Parenting

## Abstract

**Background:**

Screen time in young children is discouraged because of its negative effects on their development. However, excessive screen media use has been rising, particularly during the global pandemic when stay-at-home mandates were placed on young children in several countries. This study documents potential developmental effects of excessive screen media use.

**Method:**

This is a cross-sectional study. Participants were 24 to 36 month old Filipino children recruited through non-probable convenience sampling from August to October 2021. Regression analyses were performed to test the association between screen time and changes in scaled scores for skills and behaviors determined from the Adaptive Behavior Scale and to identify factors associated with increased screen media use.

**Results:**

Increased odds of excessive use of screen media of children by 4.19 when parents watch excessively and 8.56 times greater odds when children are alone compared to watching with a parent or other children. When adjusted for co-viewing, more than 2 h of screen time is significantly associated with decrease in receptive and expressive language scores. The effects on personal skills, interpersonal relationships and play and leisure skills were only statistically significant at 4 to 5 or more hours of screen time use.

**Conclusion:**

The study found that spending no more than 2 h screen time had minimal negative effects on development and that use beyond 2 h was associated with poorer language development among 2 year olds. There is less excessive screen media use when a child co-views with an adult, sibling or other child and when parents likewise have less screen time themselves.

**Supplementary Information:**

The online version contains supplementary material available at 10.1186/s12889-023-16188-4.

## Background

The Philippines is among the top media users worldwide, and online activities have been increasing through the years [1]. With a population of 110 million Filipinos, there were 73.91 million internet users in the Philippines recorded in January 2021 with 92% connected with a smartphone, 74% through a laptop or desktop computer and 38% with a tablet [2]. Globally, there is also an increase in trend for subscription-based services for entertainment viewing and the Asia Pacific countries are also growing in the use of online gaming [3].

Increasing availability of technology worldwide has inevitably increased exposure to screen media for young children, potentially affecting their development. The prevalence of excess screen time has ranged from 10 to 98% of children with 0.1 to 5 h of screen exposure per day with documented increase during the COVID-19 pandemic [4, 5]. Data examining screen media use from Western countries is growing, but there is a gap in literature measuring the effects of media exposure in other populations, including Filipinos [6].

The relationship between screen time and the cognitive development of children is complex and has had both positive and negative outcomes [7]. Excessive screen time use in young children has been associated with risks for developmental delays, attention problems, and poorer academic performance [8–10]. Extended screen time periods and its potential disruption in the basic body rhythms of children, including the circadian and eating cycles may lead to effects in sleep and nutrition [11]. There is also evidence that more screen time has been associated with unhealthy diets, obesity and poorer quality of life in children and eventually adolescents [12, 13].In contrast, joint media engagement and age-appropriate, well-designed content have shown positive associations [6, 14].

The American Academy of Pediatrics (AAP) recommends limiting screen exposure for young children to 30 min and up to 2 h depending on their age group, encourages daily physical activities and adequate sleep [15]. The World Health Organization likewise recommends no more than 1 h for children 2–5 years old [16]. However, the information regarding these recommendations may not be universally known or followed. In a study of the knowledge, attitudes and counseling practices of Filipino pediatricians, Orduña & Manalo describe the awareness of practitioners for the effects of media use in child and adolescent development, but this information is not commonly discussed with families during consultation [17]. There are parents who do not follow these recommendations and introduce children younger than 2 years old to digital media with exposures exceeding 2 h of use per day [6, 8, 18].

Infant and toddler learning from televisions and tablets has been reported, with the infant’s ability to imitate simple actions and the toddler’s ability to remember brief sequences from watching television. However, they have a transfer deficit of translating something learned from the two-dimensional television show to its application into real-life interactions, and it is easier for young children to learn from real-life interactions with people and objects compared to information delivered on a screen [6, 18, 19].

Children develop language milestones at a faster rate when supported by responsive parents in their first 2 years of life [20]. The impact of *technoference*, or the everyday interruptions or intrusions caused by devices in face-to-face interactions with others, can have detrimental effects to the relationship of parents with children, particularly when parents are distracted and miss opportunities to respond to attention-seeking cues of young children [21]. Disruption to the parent–child interaction through screen time can also have an effect on important developmental milestones [14].

Recognizing that the use of the virtual space has increased during the COVID-19 pandemic, this study aims to determine the use and effects of screen time in 2-year-old children from the Philippines. Specifically, we want 1) to measure frequency and duration of use during the lockdown period of the COVID-19 pandemic compared to professional recommendations, 2) to determine the association between use with the adaptive behavior skills of children in the domains of communication, daily living skills and socialization, and 3) to account for factors related to screen media use among children in the Philippines.

## Methods

This cross-sectional study describes demographics, quantifies the amount of screen time and describes types of screen media used by children. It also determines an association between duration of screen time and adaptive behavior skills, parent factors and environmental factors.

### Sampling population

Participants were children aged 24 to 36 months who were residing in the Philippines at the time of the study. Inclusion criteria were children residing in any region of the country. The respondents were any primary caregiver of the child. A primary caregiver was identified as someone who cares for the child regularly or has knowledge on the details of a child’s daily activities. All tools were in English and required at least 5th grade reading comprehension. The medium of education in the Philippines is taught in English and Filipino, hence a screening question for eligibility in this study required that the caregiver completed at least grade-school education. Children already diagnosed with a developmental delay or receiving interventions were excluded from the study.

Recruitment was through non-probable convenience sampling from August to October 2021 via online recruitment. There is no existing data on screen time use among children in the Philippines, and this design allowed collection from a wider range of participants nationwide. An invitation was shared through social media and through pediatricians’ clinics for patients who meet the inclusion criteria of the study. Continuous recruitment was done for 2.5-months using Survey Monkey as an online platform, which screened for eligibility for the study. Those who were eligible were given access to read, ask questions and provide consent. Consent was through input of the parent’s email address, name, relation to the child and submission of the form. All those who consented to participate were subsequently given an online form on demographics and other factors, the SCREENS-Q: Screen Media Use and Practices and a digital version of the Adaptive Behavior Scale.

We opted to calculate a minimum sample size requirement for the study to be able to have sufficient power to detect small but meaningful differences in behavior scores. We utilized the mean behavioral score of 10 and standard deviation of 3 based on the Adaptive Behavior Scale [23]. A total of 286 participants is required to be able to detect a 1 unit difference in scores at 80% power and alpha level 0.05. At the end of the recruitment period, 419 had completed the data collection tools.

### Study variables and data collection tools

Data collection tools were in English and included a questionnaire for demographics, questions adapted from the SCREENS-Q: Screen Media Use and Practices, and the Adaptive Behavior Scales.

#### Demographics

After obtaining consent from a child’s parent or guardian, data was obtained on their background demographics, which included characteristics, socioeconomic status and parents’ background and education.

#### Screen time frequency and duration

Screen time use was based on the frequency and duration with different screen media devices such as television, mobile devices, tablets, computers, and game consoles.

In addition to demographic background information, child factors related to duration of sleep, duration of physical activity, and context of screen media use were obtained. Family factors were gathered based on parents’ practices and perceptions and rules implemented at home. Parents’ practices are related to the parent’s own duration and frequency of screen time, background screen media, and work duration. Parents' perceptions are related to their view of the child’s behaviors and learning opportunities with screen time. Rules implemented at home were based on the agreement of the parent to have fixed boundaries for screen time and how media is used such as asking permission, duration and content played or watched.

The SCREENS-Q survey developed by Klakk et. al, is a parent survey documenting screen media use of children and their parents. The questionnaire covers six domains validated as important factors of screen media and comprises 19 general questions with 92 items. Test–retest reliability ranged from 0.67 to 0.90. Kappa values were above 0.50 with more than 80% of values above 0.61 indicating good test–retest reliability. Internal consistency between different times showed good correlation from 0.59 to 0.66. The response time was recorded to not exceed 15 min [22]. This tool has not yet been used in the Philippines, therefore pilot testing of the questionnaire was done with 5 parents. Feedback was collected regarding the format of the questionnaire, readability and ease of understanding, and suggestions on how to improve the questionnaire. The questionnaire was reviewed and adapted by removing questions parents found unrelated to their children. These modifications were specifically for questions about media use in the school that are more appropriate for children that are preschool and grade-school aged. Considering the available online-based programs for children during the COVID-19 pandemic, a question on whether a child is enrolled in an online play or preschool program was retained. The modified questionnaire comprised 12 questions with 80 items. The revised questionnaire was pilot tested to an additional 5 parents. No additional recommendations were made and therefore the study was implemented.

The information gathered using the SCREENS-Q measured frequency of each item in the following categories: screen media environment, child’s screen media use, context of screen media use, early exposure, parental perception of child’s media use and parental use.

The Adaptive Behavior Scale (ABS) was used to measure the scaled scores of a child’s adaptive behaviors in areas of development relative to same-age peers. It is a tool derived from the Vineland Adaptive Behavior Scales, 3rd ed. for administration to children 16 days to 42 months old. These involve day-to-day things that a child can do to communicate, take care of themselves and get along with others. The ABS includes five subtests: Receptive, Expressive, Personal, Interpersonal Relationships and Play and Leisure [23].

The measures of ABS reflect a high degree of reliability in internal consistency (0.91 to 98), test–retest (0.72 to 0.87) and inter-rater (0.67 to 0.81) reliability. Scores for developmental delay have an accuracy score of 0.82 and language-based concerns ranged from 0.8 to 0.86. Traditional benchmarks for accuracy suggest that values of 0.80 to 0.89 are good. The tool has undergone validity and reliability testing with the study population of the Bayley Scales for Infant and Toddler Development, 4th ed. which included different ethnic backgrounds and has been a tool used in clinical assessments and research purposes. Scaled scores represent a child’s performance on a subtest relative to his or her same-age peers. They are derived from the total raw scores on each subtest and are scaled to a metric with a range of 1–19, with a mean of 10, and an SD of 3. Thus, a subtest scaled score of 10 reflects the average performance of a given age group. Scores of 7 and 13 are equivalent to 1 SD below and above the mean, respectively, and scaled scores of 4 and 16 are equivalent to 2 SDs from the mean. The sum of scores from each section is computed and converted to scaled scores from 1 to 19 with corresponding descriptive classifications. Scores of 1–3 are extremely low, 4–5 are very low, 6–7 are low average, 8–11 are average, 12–13 are high average, 14–15 are very high and 16–19 are extremely high [23].

### Statistical analysis

All analyses used STATA version 14.2 (Statacorp, College Station, TX). Adjusted linear regression was performed to test for associations between each of the five scaled scores (outcome) with duration of screen time use (main predictor), adjusting for co-viewing (defined as 1 = viewing with parent or another adult, 2 = viewing with siblings or 3 = viewing alone). In a sensitivity analysis, the same adjusted linear regression was performed for each scaled score with the addition of sex, physical activity, sleep duration, and mother’s age as covariates. To determine factors associated with increased screen time use, unadjusted logistic regression was performed for screen time use (one hour or less vs. greater than 1 h), following the World Health Organization recommendation of 1 h or less of screen use for children 2–5 years old [24]*,* with each identified child-related and family-related factors. A *p*-value < 0.05 was considered significant.

### Covariates

To determine covariates for inclusion into the regression models for the sensitivity analysis, the following variables were tested for association with each of the five scaled scores (Receptive Language, Expressive Language, Personal, Interpersonal Relations, Play and Leisure): 1) categorical variables sex and physical activity (3 h or less vs. greater than 3 h) and 2) continuous variables age (in months), sleep duration, mother’s age and father’s age. A *p*-value < 0.05 for association with any of the scaled scores was considered significant and the covariate was included in all models.

## Results

A total of 419 children were included in this study with a mean age of 28.9 (SD of 3.7) months old. There were 217 males (52%) and 202 females (48%). The parent questionnaires were predominantly answered by mothers. Majority of the mothers were college graduates (65.5%) or postgraduate degree holders (23.5%). The place of residence of participants were divided into the 3 major geographical regions of the country including the capital, namely Luzon (36.3%), Visayas (8.6%) Mindanao (5.1%), and the National Capital Region (49.9%). 335 of respondents reported residence in urban cities (80.0%) (Supplement Table 1).

**Table 1:** Access to digital devices each week in percentage. (*n* = 419)

	Frequency n(%)			
Type of device (*n* = 419)	None	Once a week	Several times a week	Almost daily
Laptop	263 (62.8)	75 (17.9)	56 (13.4)	25 (6.0)
Desktop	364 (86.9)	27(6.4)	21 (5.0)	7 (1.7)
Tablet	207 (49.4)	40 (9.6)	53 (12.7)	119 (28.4)
Smartphone	59 (14.1)	52 (12.4)	93 (21.2)	215 (51.3)
Television	43 (10.3)	40 (9.6)	78 (18.6)	258 (61.6)
Gaming console	411 (98.1)	7 (1.7)	1 (0.2)	-
Handheld gaming device	407 (97.1)	8 (1.9)	2 (0.5)	2 (0.5)
E-reader	404 (96.4)	8 (1.9)	5 (1.4)	1 (0.2)

### Frequency and duration of screen media use

Children have access to multiple screen media devices (Table 1). They are mostly using televisions (89.7%), smartphones (85.9%), and tablets (50.6%). Only 13.1% of children had experience with a desktop computer and less than 3% of children were using any game console or electronic readers.

Table 2 shows descriptive statistics of duration and content of media used during weekdays and weekends, which were similar. The recommendation for screen time in 2-year-old children is no more than 1 h, and only 99 out of 419 (23.6%) children are within the recommendation. Video calls are a frequently consumed media content with 309 (73.7%) children having access.

**Table 2:** Children’s daily media use on weekdays and weekends by type of activity in percentage per time category according to professional recommendation of 1 h of screen time. (*n* = 419)

	Weekdays—Frequency n(%)		Weekends—Frequency n(%)	
Type of media	≤ 1 h	> 1 h	≤ 1 h	> 1 h
School-related activities	406 (96.9)	13 (3.1)	414 (98.8)	5 (1.2)
Entertainment (shows and clips)	95 (22.7)	324 (77.3)	100 (23.9)	319 (76.1)
Games	364 (86.9)	55 (13.1)	375 (89.5)	44 (10.5)
Video calls	387 (92.4)	32 (7.5)	390 (93.1)	29 (6.9)
Social media	415 (99.0)	4 (1.0)	416 (99.3)	3 (0.7)

### Adaptive behavior scaled scores

The average performance of the children in this study (Table 3) show scores slightly below the mean of the normed scales of the Adaptive Behavior Scale, which has a mean of 10 with an SD of 3 [23].

**Table 3:** Descriptive statistics for 5 subdomains of the adaptive behavior scales

Adaptive Behavior Domain	Mean	Median	Mode	Standard Deviation
Receptive Language	9.95	10	11	3.59
Expressive Language	9.52	10	12	3.65
Personal	8.97	9	8	2.64
Interpersonal Relationship	9.98	10	9	2.77
Play and Leisure	9.65	10	12	3.11

### Association of screen media use with adaptive behavior skills

**Fig. 1:**
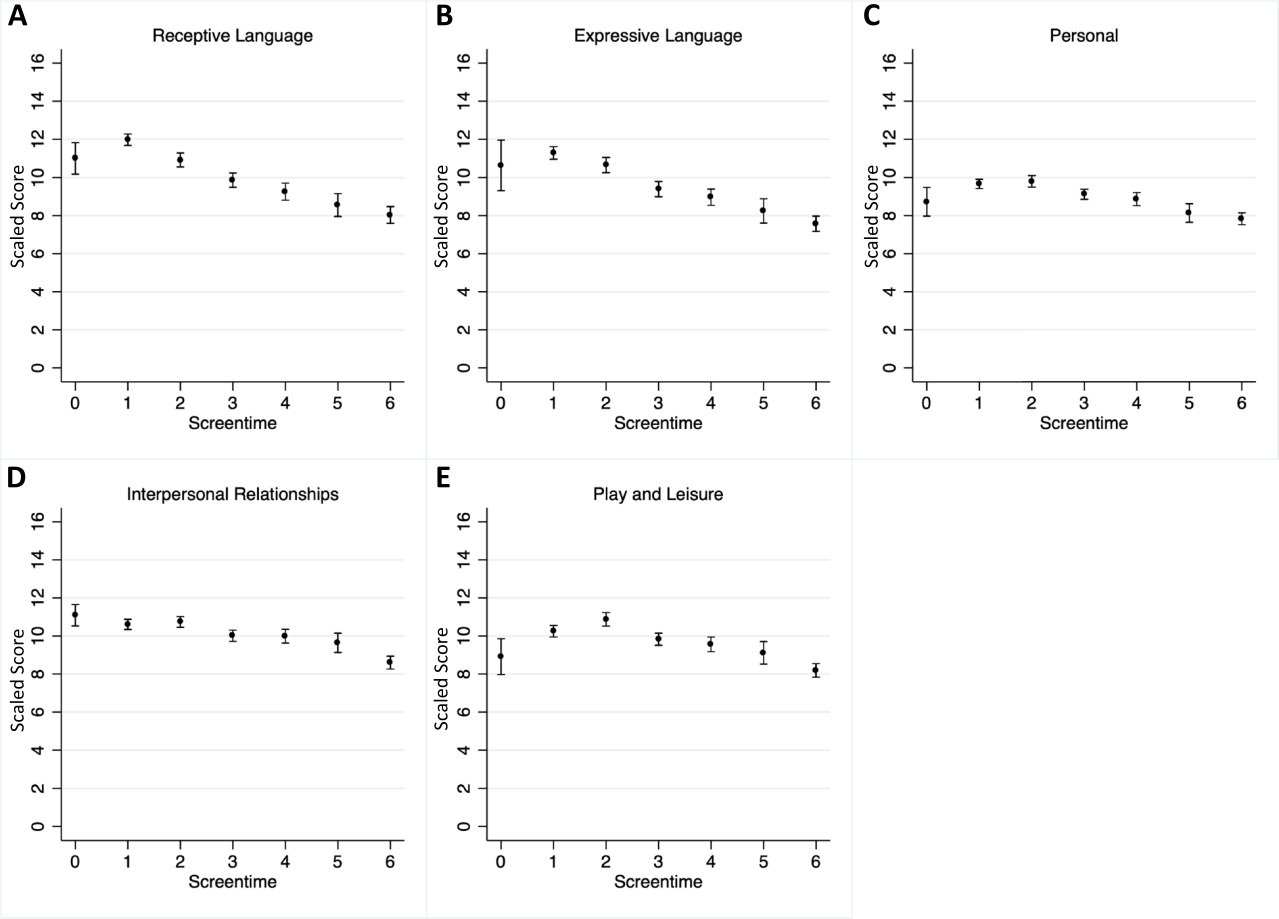
Effect of screen time use on scaled scores. Mean ± SEM of **A**) receptive language, **B**) expressive language, **C**) personal, **D**) interpersonal relationships, and E) play and leisure scaled scores by screen time use. (Screen time use: 0 = none, 1 = less than 1 h, 2 = 1 to 2 h, 3 = 2 to 3 h, 4 = 3 to 4 h, 5 = 4 to 5 h, 6 = more than 5 h)

As screen time hours increase, an associated decreasing trend in scores is observed in receptive and expressive language, personal and interpersonal skills and play and leisure skills, likened to a dose response (Fig. 1).

Adjusting for co-viewing, more than 2 h of screen time is significantly associated with decrease in receptive and expressive language scores (Table 4). More than 4 h of screen time use had significant negative effects on personal skills, while more than 5 h of screen time use had significant negative effects on interpersonal skills and play and leisure skills (Table 4).

**Table 4:** Main analysis. Estimates of linear regression of each adaptive behavior scaled score with screen time use, adjusted for co-viewing

	**Receptive**		**Expressive**		**Personal**		**Interpersonal**		**Play and leisure**	
**Screen time use**	β coefficient (CI)	*p*	β coefficient (CI)	*p*	β coefficient (CI)	*p*	β coefficient (CI)	*p*	β coefficient (CI)	*p*
None	-0.743 (-2.81 to 1.32)	0.480	-0.501 (-2.63 to 1.63)	0.644	-0.753 (-2.34 to 0.84)	0.352	0.646 (-1.02 to 2.31)	0.447	-1.120 (-2.99 to 0.75)	0.240
< 1 h	ref	-	ref	-	ref	-	ref	-	ref	-
1 to 2 h	-0.893 (-1.93 to 0.15)	0.092	-0.454 (-1.53 to 0.62)	0.405	0.223 (-0.58 to 1.02)	0.584	0.268 (-0.57 to 1.11)	0.531	0.696 (-0.24 to 1.64)	0.147
2 to 3 h	-1.957 (-2.98 to -0.93)	** < 0.001**	-1.729 (-2.79 to -0.67)	**0.001**	-0.466 (-1.26 to 0.32)	0.247	-0.469 (-1.30 to 0.36)	0.267	-0.352 (-1.28 to 0.58)	0.457
3 to 4 h	-2.449 (-3.54 to -1.36)	** < 0.001**	-2.085 (-3.21 to -0.96)	** < 0.001**	-0.631 (-1.47 to 0.21)	0.139	-0.419 (-1.30 to 0.46)	0.350	-0.521 (-1.51 to 0.46)	0.299
4 to 5 h	-3.035 (-4.33 to -1.74)	** < 0.001**	-2.755 (-4.09 to -1.42)	** < 0.001**	-1.244 (-2.24 to 0.25)	**0.014**	-0.689 (-1.73 to 0.36)	0.196	-0.816 (-1.99 to 0.35)	0.171
More than 5 h	-3.581 (-4.60 to 2.56)	** < 0.001**	-3.400 (-4.45 to -2.35)	** < 0.001**	-1.600 (-2.38 to 0.82)	** < 0.001**	-1.732 (-2.55 to 0.91)	** < 0.001**	-1.824 (-2.75 to -0.90)	** < 0.001**
**Co-viewing**										
With an adult	ref	**-**	ref	**-**	ref	**-**	ref	**-**	ref	**-**
With a sibling or other children	-0.497 (-1.54 to 0.54)	0.347	-0.917 (-1.99 to 0.16)	0.094	0.013 (-0.79 to 0.81)	0.974	-0.532 (-1.37 to 0.31)	0.213	0.255 (-0.68 to 1.20)	0.594
Alone	-2.893 (-4.22 to -1.56)	** < 0.001**	-2.231 (-3.60 to 0.86)	**0.002**	-2.040 (-3.06 to -1.02)	** < 0.001**	-2.136 (-3.21 to 1.06)	** < 0.001**	-2.266 (-3.47 to 1.06)	** < 0.001**

Next, we tested additional covariates that could potentially have an impact on each scaled score. We found that gender, physical activity, sleep duration and the age of the mother had significant associations with one or more scaled scores (data not shown). We therefore performed a sensitivity analysis with the inclusion of these covariates. More than one hour of screen time was significantly associated with a decrease in scaled scores for receptive language, while more than 2 h of screen time was significantly associated with a decrease in scaled scores for expressive language (Table 4). Results for scaled scores for personal, interpersonal and play and leisure skills were similar to results from the main analysis (Tables 4, 5).

**Table 5:** Sensitivity analysis. Estimates of linear regression of each adaptive behavior scaled score with screen time use, adjusted for co-viewing, gender, physical activity, sleep duration and mother’s age

	**Receptive**		**Expressive**		**Personal**		**Interpersonal**		**Play and leisure**	
**Screen time use**	β coefficient (CI)	*p*	β coefficient (CI)	*p*	β coefficient (CI)	*p*	β coefficient (CI)	*p*	β coefficient (CI)	*p*
None	-0.342 (-2.34 to 1.66)	0.737	-0.032 (-2.07 to 2.01)	0.976	-0.440 (-1.98 to 1.10)	0.576	0.853 (-0.79 to 2.49)	0.307	-0.933 (-2.78 to 0.92)	0.322
< 1 h	ref	-	ref	-	ref	-	ref	-	ref	-
1 to 2 h	-1.014 (-2.02 to -0.01)	**0.048**	-0.582 (-1.61 to 0.44)	0.265	0.179 (-0.60 to 0.95)	0.650	0.191 (-0.63 to 1.02)	0.648	0.597 (-0.33 to 1.53)	0.208
2 to 3 h	-2.132 (-3.13 to -1.14)	** < 0.001**	-1.983 (-3.00 to -0.97)	** < 0.001**	-0.493 (-1.26 to 0.28)	0.208	-0.543 (-1.36 to 0.27)	0.192	-0.458 (-1.38 to 0.46)	0.329
3 to 4 h	-2.509 (-3.56 to -1.46)	** < 0.001**	-2.113 (-3.19 to -1.04)	** < 0.001**	-0.633 (-1.45 to 0.18)	0.126	-0.465 (-1.33 to 0.40)	0.290	-0.592 (-1.57 to 0.38)	0.233
4 to 5 h	-3.249 (-4.50 to -1.99)	** < 0.001**	-2.947 (-4.23 to -1.66)	** < 0.001**	-1.326 (-2.30 to 0.36)	**0.008**	-0.857 (-1.89 to 0.17)	0.103	-1.033 (-2.20 to 0.13)	0.082
More than 5 h	-3.418 (-4.41 to -2.43)	** < 0.001**	-3.233 (-4.25 to -2.22)	** < 0.001**	-1.476 (-2.24 to -0.71)	** < 0.001**	-1.640 (-2.45 to -0.83)	** < 0.001**	-1.787 (-2.70 to -0.87)	** < 0.001**
**Co-viewing**										
With an adult	ref	**-**	ref	**-**	ref	**-**	ref	**-**	ref	**-**
With a sibling or other children	-0.481 (-1.48 to 0.52)	0.347	-0.914 (-1.94 to 0.11)	0.080	0.079 (-0.70 to 0.85)	0.842	-0.509 (-1.33 to 0.32)	0.226	0.275 (-0.65 to 1.20)	0.562
Alone	-2.886 (-4.18 to -1.59)	** < 0.001**	-2.394 (-3.72 to -1.07)	** < 0.001**	-1.971 (-2.97 to -0.97)	** < 0.001**	-2.056 (-3.12 to -0.99)	** < 0.001**	-2.164 (-3.36 to -0.96)	** < 0.001**

### Child factors related with screen media use

Sex of the child, physical activity or sleep duration was not a factor for screen time use. Of all the children in the study, 290 (69.2%) are able to participate in at least 3 h of physical activities daily following the recommendations by the World Health Organization [24]. The quality of sleep was not recorded, however the average sleep duration for both groups of children who had less than an hour and those who exceeded an hour of screen time use is 12 h of sleep (Table 6).

**Table 6:** Child factors associated with screen time use

Factor	Screen Time Use				
	≤ 1 h n (%)	> 1 h n (%)	Crude Odds Ratio	CI	*p*-value
1. Sex					
Girls	50 (24.8)	152 (75.2)	Ref.	-	-
Boys	45 (20.7)	172 (79.3)	1.3	0.80—1.99	0.327
2. Physical activity					
3 h or less	33 (25.6)	96 (74.4)	Ref.	-	-
> 3 h	62 (21.4)	228 (78.6)	1.26	0.78—2.05	0.344
3. Sleep duration (hours)	12 (1.8)	12 (1.8)	0.92	0.81—1.06	0.251
4. Co-viewing					
With adult	89 (25.5)	260 (74.5)	Ref.	-	-
With siblings or other children	5 (11.4)	39 (88.6)	2.67	1.02—6.98	0.045
Alone	1 (3.8)	25 (96.2)	8.56	1.14—64.07	0.037
5. Needs permission for Screen time					
Yes	93 (23.3)	307 (76.7)	Ref.	-	-
No	2 (10.5)	17 (89.5)	2.57	0.58—11.35	0.211

#### Family factors and co-viewing

Household income, parent’s educational background, place of residence or duration of work was not a factor for excessive screen time. (Table 7) Increased odds of excessive use of screen time were seen in children whose parents 1) viewed television for more than 2 h (OR = 4.19, 95% CI: 2.46—7.13, p < 0.001), 2) browsed the web for more than 2 h (OR = 1.75, 95% CI: 1.07—2.88, p = 0.027) and 3) used social media for more than 2 h (OR = 1.84, 95% CI: 1.15—2.95, p = 0.012). Most interestingly, data shows increased odds of excessive use of screen time in children who watched alone compared to watching with a parent or adult. (OR = 8.56, 95% CI: 1.14 to 64.07, p-value = 0.037) (Table 7).

**Table 7:** Family factors associated with children’s screen time use

Factor	Screen Time Use				
	≤ 1 h n(%)	> 1 h n(%)	Crude Odds Ratio	CI	*p*-value
1. Monthly household income					
Php 76,000 or less	45 (20.9)	170 (78.1)	Ref.	-	-
> Php 76,000	36 (25.2)	107 (74.8)	0.79	0.48—1.30	0.348
2. Parent’s Screen time (> 2 h)					
Television	21 (10.7)	176 (89.3)	4.19	2.46—7.13	< 0.001
Gaming	3 (8.8)	31 (91.2)	3.24	0.97—10.86	0.056
Web Browsing	27 (16.9)	133 (83.1)	1.75	1.07—2.88	0.027
Video Calling	5 (14.7)	29 (85.3)	1.77	0.67—4.71	0.253
Social media use	34 (17.2)	164 (82.8)	1.84	1.15—2.95	0.012
3. Father’s education					
At least some college	15 (17.2)	72 (82.8)	Ref.	-	-
College degree or higher	80 (24.7)	244 (75.3)	0.64	0.34—1.17	0.146
4. Mother’s education					
At least some college	8 (17.4)	38 (82.6)	Ref.	-	-
College degree or higher	87 (23.5)	284 (76.5)	0.69	0.31—1.53	0.358
5. Type of residence					
Urban	75 (22.4)	260 (77.6)	Ref.	-	-
Rural	18 (25.4)	53 (74.6)	0.85	0.47—1.54	0.590
6. Duration of working from home					
7 h or less	35 (27.3)	93 (72.7)	Ref.	-	-
> 7 h	25 (18.9)	107 (81.1)	1.61	0.90—2.89	0.109

#### Background screen media

Overall, 180 (43%) of children had regular exposure to background screen media. There was an 11 times greater odds of excessive screen time among children exposed to background screen media compared to those who do not (p < 0.001).

#### Parent perceptions: rules and behaviors

Most parents agreed to statements of having rules or fixed boundaries related to screen media use, including the duration (92.1%), time (92.3%) and content (98.6%) for entertainment programs and type (95.9%) of games played. Several respondents in the study had parents who agreed that children would use screen media to calm down (75.4%), it is an activity done together with the parent (88.1%), and a source of pleasant conversations (75.7%). There were 357 (85.2%) who reported that the child’s screen time use was an appropriate amount for leisure time. 298 (71.1%) had children who expressed a desire to use screen media everyday with 57% reporting having conflicts with the child when the media use is being limited by the parent, and 317 (75.7%) agree that the use of screen media is predominantly sedentary. Only 104 (25%) of children have difficulty thinking of things to do if he/she is not allowed to use screen media.

#### Educational use

When asked regarding a parents’ agreement that using screen media during leisure time helps children learn to write and spell, read and calculate, 255 (60.9%), 274 (65.4%) and 242 (57.8%) agreed respectively. Additionally, a larger number, 360 respondents (86%), perceive screen media use to enhance the child’s creativity and imagination.

## Discussion

### Frequency and duration of screen media use

Technology in households is increasing worldwide leading to increased availability of screen media gadgets for children [25]. This gives rise to debates on positive and negative consequences of screen time in both research and professional organizations [8, 26–28]. The variety of media devices now available to young children include televisions, smartphones, tablets and computers. Children have easy access to devices and to different varieties of media sources, which highlights the development of children within a digital world [28].

Limits to screen time have been circulated in several countries ranging from 30 min to 2 h, with a general recommendation from the World Health Organization to limit screen time to no more than 1 h a day for young children [15, 16]. It is worth noting that the quality of shows, games and activities on the devices was not quantified. It was common for the children of this study to have more than 1 h of entertainment shows each day, coinciding with reported data that most children actually view an average of 2 h per day and that longer screen time was reported in areas with longer lockdown periods related to the COVID-19 pandemic [5, 8, 29]. Data collection tools can be updated to include examples of content consumed on the media devices. For example, when shows are categorized as educational, it would be of value to know if they are developmentally appropriate for the child’s age [30–32]. The study showed no difference in screen time on weekends and weekdays unlike previous studies, which could be due to similar day to day schedules of young children during lockdown mandates of the pandemic in the Philippines [33].

### Effect of excessive screen media use on adaptive behavior skills

Children develop language faster when supported by responsive parents within their early years, and excessive screen time is detrimental to development and behavior because of overstimulation, disorganization, dysregulation and distress [8, 26, 33–40]. These can disrupt interaction with people, which limits a child’s practice of verbal and nonverbal social exchanges [27]. This study observed decreased scores in receptive and expressive language when children had more than 2 h of screen time. Supporting the growing evidence that greater time spent on devices have been increasing the risk of later language skills in young children [32, 41–43]. Similar associations were noted in personal, interpersonal and play and leisure skills when screen time was 4–5 or more hours. In addition, unsupervised media use was significantly associated with decreased scores for all domains, independent of the number of hours of screen time.

### Child factors associated with screen media use

Children in the pandemic time had restricted activities for outdoor play [44]. The amount of physical activity of children in this study was not affected by excessive use of screens unlike other reported studies that showed increased risk for sedentary behaviors with increased screen time [37, 38, 45]. Structured sports and other physical activities may have been replaced with more unstructured play for younger children [38]. Therefore, the differences in countries reporting varying amounts of physical activity are likely multifactorial and dependent on multiple contexts. Screen time and physical activities may not always be directly related since children were meeting the recommended time for movement activities even with excessive screen time use [39].

There was no difference in the average sleep duration for children with and without excessive screen time. The average of 12 h falls within the recommended 11–14 h of sleep for 2 year old children [24].

### Parent and environmental factors associated with screen media use

#### Co-viewing

There are nearly 9 times greater odds of excessive screen time when a child is watching alone compared to watching with an adult. This finding coincides with literature describing the influence that parents may have on children’s watching behaviors. It has become widely accepted that parent involvement in watching screen media may help reduce the amount of time children spend on gadgets [46]. However, it is also very common that co-viewing is a family behavior that can continue as children grow-up. This becomes a significant consideration when establishing interventions that promote healthy behaviors among children, that can include education of parents in appropriate and supportive ways of co-viewing [46–48].

#### Modeling

The interruptions caused by devices to interactions with children can affect relationships with parents when they miss opportunities to respond to attention-seeking cues of the child [14, 22]. There is a significant increase in odds of excessive screen time in children with parents who view televisions, browse the web or use social media for more than 2 h. Parents who watched more television daily were more likely to have children who watched a greater amount of television [40, 44, 49, 50]. This makes parental screen media use a strong predictor of screen time for children in their first few years of life.

#### Background television

Children in this study exposed to excessive screen time were more likely to have exposure to background screen media. Constant television in the background affects the quality and quantity of parent–child interactions that was correlated to a reduction in communication by the parent with the child [19, 21, 51, 52]. Young children’s attention are consistently shorter while they played with their toys when televisions were playing in the background [53]. These findings suggest that disruption in both the parent’s and child’s attention can decrease the learning opportunity of conversation and play [32].

### Parent perceptions on screen media use and child behaviors

Orduña & Manalo describes that screen time use is not commonly discussed in-depth with families during pediatrician visits [11]. There are also parents who do not follow recommendations and introduce children younger than 2 years old to digital media [6, 12].

Screen use has been associated with problematic behaviors such as interference with daily routines, complaints of boredom, unhappiness without access to devices, and negative emotions after use [31]. Parents in this study recognize that screen media use of children are predominantly sedentary, needed to calm children down and develops an increased desire of children to use the screen media daily. It has also been a source of conflict when its use is being limited. However, only 24.8 percent of children would actually have difficulty finding alternative activities once the use of the screen has been limited.

Parents recognize that screen media can be the source of challenging behaviors of their children. However, potential negative effects do not necessarily translate to the practice of limiting the exposure of children to longer periods of time [54]. Majority of the parents also perceive screen time as a source for learning, creativity and imagination, which corresponded to results suggesting that children may be permitted to watch for longer periods of time. This study does not make associations between reasons for allowing extended screen time use and if there are perceived benefits or consequences. However, these findings can influence further research that possibly identifies appropriate materials and methods of delivering content to younger children [32].

### Implication to recommendations for Filipino children

There is a need to emphasize the importance of measuring quality of screen time, including content viewed and context in which screen media is used [40, 48, 55]. Based on this study, it is advisable to have no more than 2 h of screen media use with emphasis that content available must be appropriate for the child’s developmental skill level and age that can be guided by a parent through co-viewing.

The WHO recommendations on physical activity and sleep were maintained by the study cohort. Further review on factors that promoted this in spite of longer screen time, could provide further resources for parents and professionals to consider in the promotion of healthy development of children. There is also a need to investigate and highlight strategies to better support development of children and manage screen time for subsequent COVID-19 rise in cases, post-pandemic or future situations that limits the activities of young children [56].

## Limitations

The data of this study is taken from one point in time and cannot measure directionality when comparing the scores with the media use of children. However, the information provides initial information to screen time use in the Philippines to support further investigation to generate additional information. Limitations of the study include the following:

1. The assessment questionnaire was administered in English. Participants were limited to those with English reading proficiency and internet connectivity. Based on the demographic distribution, respondents were from urban areas and within the middle socioeconomic class. Therefore, the values related to screen time and developmental domain scaled scores may not be representative of other areas and sociodemographic categories of the country.
2. Screen time and developmental skills were not validated against observable measures. There are studies that report parent perception error of > 60 min compared to actual child’s use of a device [30].

## Conclusions

Television, closely followed by smartphones, is still the most common source of screen media and the majority of children have excessive screen time use based on WHO guidelines of not exceeding 1 h a day. However, when reviewing the effect on development, receptive and expressive language were significantly decreased in children spending more than 2 h of screen time. Excessive screen use was seen in children who typically watched alone and had parents who spent more than 2 h watching television, web browsing or social media.

While not exceeding 2 h of screen time would have minimal negative effects on 2-year-old development, it may be beneficial to follow the guidelines set by WHO in limiting screen time to less than 1 h for this age group. It is likewise emphasized that co-viewing with an adult, sibling or other children is recommended because children learn through modeling and interactions with others. Parents perceive various benefits that support children’s learning and this may contribute to their decision to allow more screen time. Further investigation is needed on content and quality of screen media to provide additional information to support parent education and anticipatory guidance for young children.

## Supplementary Information

**Additional file 1.** 

## Data Availability

The datasets used and/or analyzed during the current study are available from the corresponding author on reasonable request.

## Abbreviations

AAP: American Academy of Pediatrics
ABS: Adaptive Behavior Scales
CALABARZON: Region comprising the provinces of Cavite, Laguna, Batangas, Quezon, Lucena
CAR: Cordillera Administrative Region
COVID-19: Coronavirus disease caused by the SARS-CoV-2 virus
NCR: National Capital Region
OR: Odds ratio
WHO: World Health Organization
