# Supplementary material for: Measuring effects of screen time on the development of children in the Philippines: a cross-sectional study
Source: BMC Public Health. 2023 Jun 28;23:1261. doi: 10.1186/s12889-023-16188-4 (PMC10308687; doi:10.1186/s12889-023-16188-4)
Supplement: Supplementary file 1 — Additional file 1. [file 12889_2023_16188_MOESM1_ESM.pdf]

**Measuring Effects of Screen Time on the Development of Filipino Children: A Cross-sectional Study (Dy, et. al.) - Supplement Tables**

Supplement Table 1. Demographics of study population

| **Age of child (months)** | n | |
|---|---|---|
| Mean<br>Median<br>Mode<br>Range<br>± Std. Dev | 28.9<br>29<br>24<br>24-36<br>3.7 | |
| **Sex (Frequency, %)** | | |
| Male<br>Female | 217 (52%)<br>202 (48%) | |
| **Age of Parents (years)** | Mother | Father |
| Mean<br>Median<br>Mode<br>Range<br>± Std. Dev | 32.9<br>32<br>32<br>20 - 67<br>6.3 | 34.4<br>33<br>33<br>22 - 68<br>6.7 |
| **Highest Educational attainment (Frequency, %)** | | |
| None | - | 2 (0.5) |
| Some elementary school | - | - |
| Elementary school graduate | - | 4 (1.0) |
| High school graduate | 10 (2.4) | 17 (4.1) |
| Some college | 26 (6.2) | 43 (10.5) |
| Vocational course | 10 (2.4) | 21 (5.1) |
| College graduate | 273 (65.5) | 271 (65.9) |
| Post-graduate degree | 98 (23.5) | 53 (12.9) |
| **Household income (Frequency, %)** | | |
| < Php 11,000 | 13 (3.1) | |
| Php 11,000 to 22,000 | 38 (9.1) | |
| Php 22,001 to Php 44,000 | 83 (19.9) | |
| Php 44,001 to Php 76,000 | 81 (19.4) | |
| Php 76,001 to 131,000 | 75 (17.9) | |
| Php 131,001 to 219,000 | 37 (8.9) | |

| | |
|---|---|
| > Php 219,000 | 31 (7.4) |
| Preferred not to say | 60 (14.4) |
| **Residence by Region (Frequency, %)** | |
| National Capital Region (NCR) | 208 (49.9) |
| Luzon<br>(Regions I, II, III, IV-A, V, CAR and MIMAROPA) | 152 (36.3) |
| Visayas<br>(Regions VI, VII, VIII) | 36 (8.6) |
| Mindanao<br>(Regions IX, X, XI, XII, XIII, BARMM) | 21 (5.1) |
| **Residence by Type of City (Frequency, %)** | |
| Urban | 335 (80.0) |
| Rural | 71 (17.0) |
| Unknown | 13 (3.1) |
